# Investigation of the Bleeding Tendency in Sudanese Female Carriers of Hemophilia B

**DOI:** 10.1155/2022/6756130

**Published:** 2022-06-22

**Authors:** Ismail Ali Abdallah Abker, Salaheldein G. Elzaki, Salih Abdelgader Elmahdi, Jowaria Eltayeb Tayrab, Samia Mahdi Ahmed, Eltayeb Tayrab

**Affiliations:** ^1^Department of Hematology, National Ribat University, Khartoum, Sudan; ^2^Department of Molecular Hematology, Tropical Medicine Research Institute, National Center for Research, Khartoum, Sudan; ^3^Department of Chemical Pathology, National Ribat University, Khartoum, Sudan; ^4^Wexford General Hospital, 12 Prior Court Spawell Road, Co Wexford, Ireland; ^5^Department of Medical Laboratory Sciences, College of Applied Medical Sciences, Taibah University, Medina, Saudi Arabia

## Abstract

**Background:**

Hemophilia (HB) is an X-linked, recessive bleeding disorder characterized by the deficiency or absence of the coagulation factor IX. Usually, females are carriers of the trait, while males are affected. FIX deficiency leads to uncontrollable bleeding events, and the severity is dependent on the levels of the clotting factor. The objective of this research was to measure the prevalence of bleeding tendency in Sudanese carriers of HB.

**Materials and Methods:**

In this cross-sectional study, 88 Sudanese carriers of HB participated. The activated partial thromboplastin time test (APTT) and FIX test were performed for each carrier. The frequencies of DNA polymorphism and FIX-linked restriction fragments BamHI, HhaI, and MnII were also assessed. The study was conducted in Khartoum, Sudan, during the period from 2015 to 2017.

**Results:**

The study showed that 55 (62.5%) HB carriers were from the Laban village in the White Nile State, and all of them were members of the Shinkheb tribe. The mean age of the study population was 26.3 years. Among the carriers, 57 (64.7%) had abnormal coagulation profiles. The mean value of the APTT level among carriers was significantly increased (*P* value: 0.000), while the mean concentration of the FIX levels among the carriers was significantly decreased (*P* value: 0.000). The study also showed a negative correlation between PTT and F assay with *P* value of 0.000 and *R* value of 0.578.

**Conclusion:**

The APTT is high in most carriers and the FIX assay level is low in most carriers. Most carriers had no symptoms and were not bleeding. The Shinkheb tribe is the most ethnic tribe carrying HB (62.5%). HhaII is more informative for carrier detection than others, but it is of significant value if both (MnII and HhaII) were performed in parallel. In Sudanese, BamHI was informative but MnII and HhaII were best in the mutation detection and for prenatal diagnosis.

## 1. Introduction

Hemophilia is an inherited genetic disorder that impairs the blood clot [1]. The disease affects males while female relatives are carriers [2]. It has been found that for each hemophilic male patient, there are five female carriers [3]. Hemophilia is a common severe bleeding disorder, and if not properly managed since early childhood, it may lead to chronic diseases and lifelong disabilities [4]. There is a wide variation in the prevalence of hemophilia between countries [5]. In Africa, hemophilia patients account for less than 3% [6]. Hemophilia B (Christmas disease) (HB) [7] is a very rare disease; it is an X-linked, recessive bleeding disorder characterized by the deficiency or absence of the coagulation factor IX (FIX) [1]. FIX deficiency leads to uncontrollable or abnormal bleeding events, and the frequency and severity are dependent on the clotting factor levels [8] & [1]. FIX deficiency was identified genetically in 1952 [9]. HB is a result of a heterogeneous mutation in the FIX gene (*F9*) leading to a defective coagulation process [10]. Females are carriers of the trait, while males are affected [11]. Two types of carriers were identified: obligatory and possible carriers [12], and most of the carriers are asymptomatic in their daily life [12]. Recently, some females have been reported to be hemophilic and may have complex genetic causes for their hemophilia phenotype [13]. Even the global rate of HB is less than that of hemophilia A (1 : 5) [8], & [11]; HB occurs in approximately 1 in 20,000–30,000 live male births [2]. Globally, around one-third of HB patients have a moderate-severe disease [14]. According to the FIX levels, HB is classified into three types: mild (5%–40%), moderate (1%–5%), and severe (less than 1%) [15]. Patients with mild to moderate types of HB may have bleeding episodes, while those with a severe form have experienced spontaneous bleeding into muscles and/or joints [16].

Carriers with clotting factors below 60% may experience bleeding tendency, and symptoms and bleeding correlate with the factor deficiency [17], & [12]. In most cases, the carrier females may have increased bleeding during menstruation and after delivery [17], & [12]. In neonates with intracranial hemorrhage or bleeding post circumcision, hemophilia is suspected, and clear female sex does not exclude the disease [18], & [13]. Carriers with lowered clotting factor levels, especially pregnant carriers, may have a higher risk of postpartum bleeding [19]. The bleeding rates in adults and children having HB are similar [20]. Hemophilic females may have excessive bleeding, because they have heterozygous alleles for hemophilia [13]. One-quarter of mild hemophilic females have HB [13]. The genes responsible for the synthesis of FIX are located on the X chromosome [4].

The hemophilia trait in females is recessive because a normal X chromosome is also responsible for at least a 50% level of the coagulation factor FIX [1], & [4]. If a hemophiliac male with one abnormal X chromosome and a healthy female with two normal X chromosomes have children, all of their daughters will be hemophilia carriers, while all of their sons will be healthy [4], & [1]. Carrier women with reduced FIX activity levels may also experience some bleeding [21]. Carrier analysis is required in a female relative, when a male relative is diagnosed to have hemophilia or when the female got pregnant [10]. The carrier women with HB may develop inhibitory antibodies, especially in those with large deletions [21]. In contrast, if a woman who is a hemophilia carrier has children from a healthy man, a boy child is at 50% risk of being affected and a girl child is at 50% risk of being a carrier of hemophilia [4], & [1].

In developing countries, hemophilia is an economic burden for its related morbidity and mortalities [22]. Coagulation factor replacement therapy is the typical hemophilia treatment either prophylactically or on-demand [23].

The objective of this study was to measure the prevalence of bleeding tendency in Sudanese carriers of HB, by assessing the levels of APTT and FIX. The frequencies of DNA polymorphism and FIX-linked restriction fragment length polymorphisms (RFLPs), which were BamHI, HhaI, and MnII, had been studied in Sudanese families with HB for carrier detection and prenatal diagnosis (PND).

## 2. Materials and Methods

### 2.1. Demographic Data

This cross-sectional study was carried out in Khartoum, Sudan, from 2015 to 2017. In this study, 88 HB carrier females participated. Of them, 55 were from White Nile Province (Laban village) about 7 km south-west of the Kosti town, the capital of the province, 20 were from Omdurman, 9 were from Bahry, and 4 were from the Khartoum town.

All patients were registered in the Khartoum Hemophilia Treatment Center.

### 2.2. Methodology

Under sterile conditions, 2.5 ml of venous blood was taken from each individual and delivered to a vial containing trisodium citrate anticoagulant. Platelet-poor plasma (PPP) was prepared by centrifugation at 2000 g for 15 min at 4°C (approximately 400 rev/min in a standard bench centrifuge). A prompt coagulation study including the activated partial thromboplastin time (APTT) test and FIX assay was performed for each carrier according to the study of [24]. The study included 9 Sudanese families.

### 2.3. APTT Measurement

Using a coagulometer adjusted at 37°C, 0.1 ml of a mixture of phospholipids and Kaolinreagent was added to a glass tube. Then, 0.1 ml of plasma was added, mixed, and incubated for 3 min, and then, 0.1 ml of prewarmed CaCl2 was added and the stopwatch was started. The time taken for the mixture to clot was recorded. The normal range of APTT by this method was typically 35–45 s.

### 2.4. Method for FIX Assay

In the coagulometer using the same APTT method mentioned above, 0.1 ml of FIX reagent was added to a mixture of 0.1 ml of APTT reagent and 0.1 ml of PPP, mixed again, and then incubated for 3 min. Finally, 0.1 ml of CaCl2 (0.025 mol/l) was added. Then, the result was recorded. The normal range of FIX according to this method was 70–120 IU/ml.

For gene analysis, real-time polymerase chain reaction (RT-PCR) was used.

### 2.5. DNA Extraction

DNA was extracted by using 300 *μ*l of carrier blood samples, collected in a trisodium citrate container; about 300 *μ*l was added to a volume of 1.5 ml Eppendorf tube containing 900 *μ*l of red blood lysing buffer, then mixed thoroughly by vortexing, incubated for 5 min at room temperature (RT), then inverted again twice during the incubation period, and centrifuged at 12000 rpm for 1 min. The obtained supernatant was removed and the white pellet was precipitated (~100 *μ*l). The pellet was vortexed strongly to resuspend the cells, 300 *μ*l of the lysed cells was added to the resuspended cells, 1.5 *μ*l of RNase was added and incubated for 15–30 min at 37°C, and the tubes were inverted many times. Samples were chilled to RT and 100 *μ*l of a protein precipitating buffer was added to the cell lysate and vortexed at high speed for 20 s. Then, samples were centrifuged at a high speed (13000 rpm) for 5 min to form a tight white pellet. A 300 *μ*l of the supernatant from each tube was transferred into the 1.5 ml tube (leaving the principate protein pellet), and 300 *μ*l of 100% isopropanol solution and the samples were mixed by inverting gently several times. Then, the samples were centrifuged vigorously at 13000 rpm for 1 min. Then, the supernatant was poured off and the tubes were drained by using cleaned absorbent paper. Next, 1 ml of 70% ethanol alcohol was added, inverted shortly several times to wash the DNA pellet, and then centrifuged at 13000 rpm for 1 min, the ethanol alcohol was poured off carefully, and the tubes were inverted and drained on the clean absorbent paper and left to dry for 10–15 min. The rehydrated buffer (150 *μ*l) was added to each tube and incubated at 65°C for about 10 min.

### 2.6. DNA Quantification

Extracted DNA (10 *μ*l) was added to 90 *μ*l of free water nuclease, vortexed, and kept at RT for 10 min for homogenizing the DNA solution, using absorbance at 260 nm, and the DNA was quantitated. An absorbance ratio of 260 nm to 280 nm estimated the purity of the solution, and the ratio was in the range of 1.5–1.9 (Gene Quant pro From Biochrom, UK). Then, the samples were stored at −20°C until the PCR amplification.

### 2.7. Amplification 3' of the FIX Gene

Each of the downstream and upstream primers was prepared as follows: to 90 *μ*l of PCR water, 10 *μ*l of each stock primer (100 *μ*M) was added and aliquoted in a 0.5 ml PCR polypropylene tube to give a concentration of 10 *μ*M, and the solution was mixed very carefully using sterile tips to ensure the homogeneity. The extracted DNA was brought from −20°C, then thawed, and kept on an ice cryo-rack for processing. Meanwhile, stock primers and ready mixed were then brought to RT and kept on the ice cryo-rack for thawing. Then, the sterile PCR water was brought out from the refrigerator and aliquoted on a 1.5 ml tube and then maintained as above. The PCR was carried out as described in the instructions, and 5 *μ*l of extracted DNA was added into 13 *μ*l of the master mix containing Mgcl2 at a concentration of 4 mM, dNTPs at 400 *μ*M, 2 units concentration of Taq DNA polymerase, PCR buffer, and dye. Into the Bio-Rad thermocycler, the mixture was loaded for the flowing temperature profile of initial denaturation at 94°C for 3 min, then denaturation at 94°C for 30 s, annealing at 58°C for 30 s, extension at 72°C for 30 s, and final extension at 72°C for 3 min, and then it was held at 4°C.

### 2.8. Visualization of PCR Products

To 1× Tris–borate–EDTA (Tris–borate pH 8.0 and 0.002 M EDTA pH 8.0), 1.5% agarose was added. Then, the gel was stained with ethidium bromide (0.5 mg/ml). Then, 5 *μ*l of the PCR products was added into each gel well, along with a 100 bp molecular weight marker (from Vivianites, Singapore), which was used for comparison of the band size, and then, the gel was run for 30 min at 80 V/cm and then viewed on an ultraviolet transilluminator.

### 2.9. Restriction Enzymes

The selection of amplified products for digestion by retraction enzymes (BamH, MnII, and Hha1) was done according to the manufacturer's protocol. That was done by adding five units of the enzyme (BamH, MnII, and HhaI) to 2 *μ*l of 10× buffers with BSA 10 *μ*l of the PCR products which were added into 7.5 nuclease-free water, then mixed, and incubated for16 h at 37°C, and the reaction was stopped at 65°C for 20 min. In restriction enzymes in gel loading, we used ladder as control.

## 3. Results

**Figure 1:**
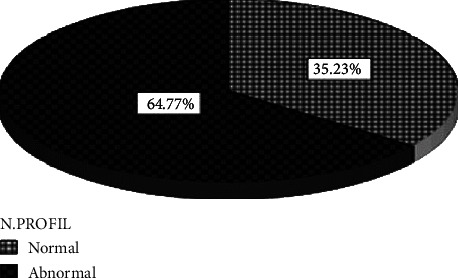
Normal and abnormal coagulation profiles in hemophilia B carriers in the study population.

The study showed that 55 (62.5%) HB carriers were from the Laban village and all of them belong to the Shinkheb tribe, 20 (22.7%) were from the Omdurman City, 9 (10.2%) were from the Bahri City, and finally 4 (4.6%) were from the Khartoum city. The mean age of the study population was 26.26 years, ranging from 3 to 65 years. The study also showed that the coagulation profile in the carriers (57 (64.77%)) showed abnormal APTT with mutation (Figure 1), whereas 31 (35.23%) carriers had a normal coagulation profile and had the mutation. The mean value of the APTT level among carriers was significantly increasing (*P* value: 0.000) (Table 1).

**Figure 2:**
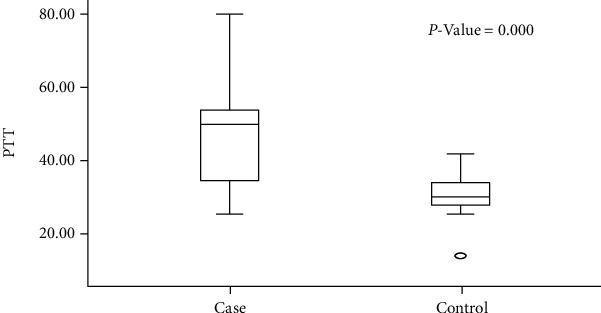
Means of PTT levels among hemophilia B carriers and their controls (*n* = 176).

The study also showed that the coagulation profile in the carriers (31 (35.23%)) was with normal FIX assay and showed a mutation. On the other hand, 57 (64.77%) carriers had abnormal coagulation profiles and had mutation (Figure 1). The mean value of the APTT level among carriers was significantly increasing (*P* value: 0.000) (Figure 2). In the control group, values of APTT and FIX levels were in the normal ranges.

**Figure 3:**
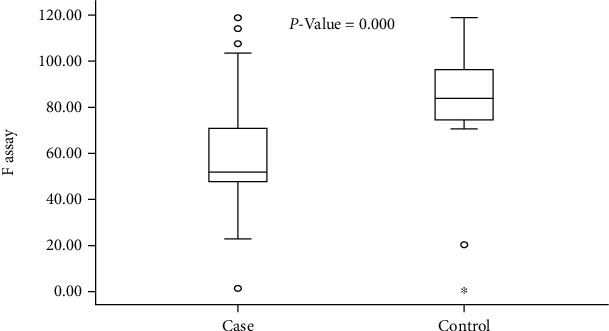
Means of FIX assay level among hemophilia B carriers and their controls (*n* = 176).

As shown in Figure 3, the mean FIX level among carriers was significantly decreased with a *P* value of 0.000 in comparison with the controls.

**Figure 4:**
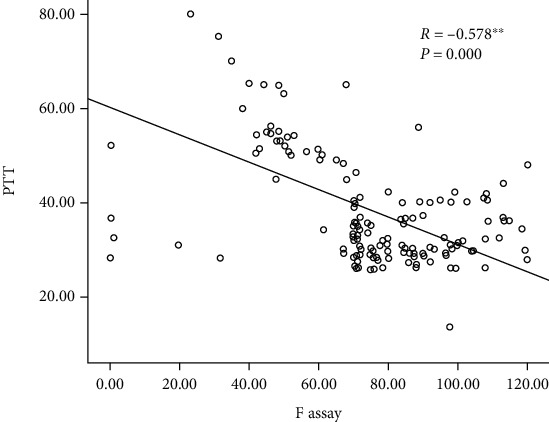
The Pearson correlation between the PTT and F assay.

Concerning the ethnic groups included in the study, the most Sudanese tribe with HB carriers was found to be the Shinkheb tribe with a frequency of 55 (62.5%), followed by the Zaghawa tribe and Awlad Rashid (A. Rashid) tribe. The Pearson correlation between the PTT and FIX assay showed a negative correlation between the PTT and FIX assay, with a *P* value of 0.000 and an *R* value of −0.578 (Figure 4).

In the digestion product BamH, the digested allele was in 379 bp and 146 bp fragments, while the allele without the recognition site for the extracted DNA enzyme remained the same (526 bp).

In the case of the digestion product of the restriction enzyme MnII, the digested allele results were 120/158 bp fragments and 278/120/158 bp fragments, while the allele without the recognition site remained the same as 278.

In the case of the digestion product of the restriction enzyme HhaII, the digested allele results in 150/80 bp fragments and 230/150/80 bp fragments, while the allele without the recognition site remained the same as 230 bp. (Table 2). The mutation 3' of the FIX gene appeared to be homozygous (++), which was inherited from both parents, or heterozygous (+−), which was inherited from either their fathers or mothers (Table 1).

## 4. Discussion

Hemophilia in Sudan is considered one of the rare diseases that are underdiagnosed or neglected and thereby undertreated. Ethnic predisposition and geographic distribution for HB may be identified in Sudan. In the current study, 62% HB carriers were from the Laban village in White Nile State, where a significantly higher frequency of HB in some studied tribes was found. The predominant ethnic group was Shinkheb, which is known to have a high rate of consanguinity marriage among populations. These findings are inconsistent with that reported by [25] in Pakistani people. Sudanese carriers with FIX deficiency experience a high rate of bleeding tendency and an abnormal coagulation profile as shown in Figure 1. Approximately 62% of carrier females had prolonged APTT levels (Figure 2), which are in accordance with the findings of [21]. The risk in these carriers is that after receiving anaphylaxis treatment, they may develop inhibitory antibodies, which may lead to the nephrotic syndrome as reported by [21].

In this study, the concentration of FIX assay levels among HB carriers significantly decreases (FIX level: 35%) (Figure 3), with a *P* value of 0.000 when compared to their controls; this is in agreement with a study carried out by [12].

In this study, most of the carriers have an abnormal coagulation profile (high PTT and low FIX level (64%)) and some have a normal coagulation profile and a normal FIX level (35%), but the normality would never necessarily indicate a noncarrier. These findings are in agreement with those of [26], who found that moderately severe or severe deficiency of clotting activity markedly prolonged with the thromboplastin time. The variation between symptoms and observed signs among HB carriers may be due to gene defects in this research. In this study, PTT was significantly prolonged in HB carriers compared to the controls (*P* value = 0.000). The mean PTT level among HB carriers and their controls was 64.7 s, and the plasma correction was normal which indicates FIX deficiency. These abnormalities of PTT and F1X assay correlate with BamH, MnII, and HhaII enzyme cutting mutations; normal parameters also have a cutting mutation with these enzymes (Table 3). BamH has positive and negative cutting with PTT and F1X assay, but MnII and HhaII have positive cutting mutation only with the PTT and F1X assay.

Hence, MnII and HhaI may be the best predictors of mutation in HB carriers and PND.

This study showed that the allele frequency of the RFLP markers showed remarkable heterogeneity among different ethnic groups. MnII proved to be the most efficient marker in the A. Rashid group with a homozygous allele (+) of 88% than other groups, but there is no statistically significant difference (*P* > 0.05, chi^2^ 10.41). As shown in Table 1, MnII is similar to BamHI with allele (+) in the Turgm tribe. Regarding the BamHI enzyme, it is the most frequent marker in the Tergum tribe with allele homozygous (+) (67%), and this difference is statistically significant (*P* < 0.05, chi^2^ 27.73). As shown in Table 1, in the HhaI marker, the higher allele (+−) was found in the Shinkheb and Zaghawa tribes with nearly similar percentage as 65% and 64%, respectively, with a highly statistically significant difference (*P* < 0.001, chi^2^ 30.09).

In this research, the highest ethnic group with HB carriers is the Shinkheb tribe with 55 (62%), who live in the White Nile State in the Laban village, which is about 7 km away from the Kosti town at the west south site, and the second ethnic group is the Zaghawa tribe, followed by A. Rashid, Batheen, and other tribes including Turgm, Arakia, Turkish, and Shooluk, respectively, according to the prevalence of hemophilia among the tribes. In Sudan, there is a wide difference in the prevalence of HB within the tribes and ethnic groups, some of them are Arabs (Shinkheb and A. Rashid) and other Necro groups (Zaghawa). It seems that the genetic causes in Sudan are strongly rooted as reported by [5], which is also reported earlier in Italy by [10].

HB carriers may experience episodes of bleeding following any trauma, menstrual cycle, delivery, and tooth extract where the collaboration of the dentists with the hematologists is required as stated by [27]. These episodes may directly affect their quality of life as reported in the United States by [15].

This means that prompt and accurate diagnosis of the HB patient's relatives, both the first and the second degree, is required for the provision of life-saving care. The second and the most important one is to avoid endogamy among the Sudanese carriers and hemophilic patients because it is a pick for community health destruction.

## 5. Limitations

The bleeding score and prenatal study in the Sudanese carriers of hemophilia B were not assessed in this research.

## 6. Conclusion

APTT is high in most carriers and FIX assay is low in most carriers. Most carriers were not complaining and were not bleeding. The Shinkheb tribe is the most ethnic tribe carrying HB (62.5%). HhaI is more informative for carrier detection than others, but it is significant if both were done parallel. BamH is informative and MnII and HhaII are best in mutation detection and for PND in Sudan. Hemophilia B is found with high prevalence in some Sudanese ethnic groups, not as thought previously that hemophilia is not common in Sudan. More research with big sample size is required to solidate the obtained results.

## Figures and Tables

**Table 1:** Allele frequency of RFLP markers in Sudanese population of hemophilia B carriers.

Tribe	BamHI			MnII		HhaI		
	+	+−	−	+	+−	+	+−	Total
A. Rashid	0.00%	25.0%	75.0%	87.5%	12.5%	100.0%	0.00%	100.0%
Tergum	66.7%	33.3%	0.00%	66.7%	33.3%	0.00%	100.0%	100.0%
Batheen	20.0%	0.00%	80.0%	20.0%	80.0%	0.00%	100.0%	100.0%
Hwezma	0.00%	100.0%	0.00%	0.00%	100.0%	0.00%	100.0%	100.0%
Erekia	50.0%	50.0%	0.00%	50.0%	50.0%	0.00%	100.0%	100.0%
Turkish	17.3%	50.0%	32.7%	51.9%	48.1%	32.7%	67.3%	100.0%
Shooluk	0.00%	100.0%	0.00%	100.0%	0.00%	0.00%	100.0%	100.0%
Zaghawa	50.0%	35.7%	14.3%	64.3%	35.7%	35.7%	64.3%	100.0%
Shinkheb	22.7%	44.3%	33.0%	55.7%	44.3%	35.2%	64.8%	100.0%

Note: + homozygous, +− heterozygous, and − negative.

**Table 2:** APTT, FIX assay, and FIX alleles in hemophilia B carriers of the study population.

Enzymes	APTT (s) (*mean* ± *SD*)	FIX assay (U/ml) (*mean* ± *SD*)	Frequency (%)	Genotypes	Carrier status
BamHI	45.5 ± 11.2	57.1 ± 24.9	20 (23)	AA	Carrier
	47.6 ± 13.1	63.3 ± 25.9	39 (44)	Aa	Obligatory carrier
	47.4 ± 13.3	59.5 ± 18.56	29 (33)	A	Non
MnII	45.7 ± 10.9	63.2 ± 20.3	49 (56)	AA	Carrier
	48.8 ± 14.5	57.60 ± 26.6	39 (44)	Aa	Obligatory carrier
HhaII	47.5 ± 11.6	61.4 ± 19.8	31 (35)	AA	Carrier
	46.8 ± 13.4	60.3 ± 25.25	57 (65)	Aa	Obligatory carrier

**Table 3:** Comparison of the bleeding profile between normal carriers and abnormal carriers.

Parameters	Normal (*mean* ± *SD*)	Abnormal (*mean* ± *SD*)	*P* value
PTT (s)	35.62 ± 6.84	53.38 ± 10.58	0.000
Correction	30.75 ± 3.47	30.95 ± 4.03	0.806
FIX assay (U/ml)	81.53 ± 22.56	49.40 ± 14.34	0.000

## Data Availability

Data are available on request.
